# Artemin Is Upregulated by TrkB Agonist and Protects the Immature Retina Against Hypoxic-Ischemic Injury by Suppressing Neuroinflammation and Astrogliosis

**DOI:** 10.3389/fnmol.2021.645000

**Published:** 2021-04-12

**Authors:** Hsiu-Mei Huang, Chao-Ching Huang, Linda Yi-Chieh Poon, Ying-Chao Chang

**Affiliations:** ^1^Department of Ophthalmology, Kaohsiung Chang Gung Memorial Hospital, Chang Gung University College of Medicine, Kaohsiung City, Taiwan; ^2^Department of Pediatrics, National Cheng Kung University Hospital, Tainan, Taiwan; ^3^Department of Pediatrics, Kaohsiung Chang Gung Memorial Hospital, Chang Gung University College of Medicine, Kaohsiung City, Taiwan

**Keywords:** Artemin (ARTN), astrogliosis, c-Jun N-terminal kinase (JNK), extracellular signal-regulated kinase (ERK), hypoxic-ischemia injury, immature retina, neuroinflammation

## Abstract

Hypoxic-ischemia (HI) is a major cause of acquired visual impairment in children from developed countries. Previous studies have shown that systemic administration of 7,8-dihydroxyavone (DHF), a selective tropomyosin receptor kinase B (TrkB) agonist, provides long-term neuroprotection against HI injury in an immature retina. However, the target genes and the mechanisms of the neuroprotective effects of TrkB signaling are not known. In the present study, we induced an HI retinal injury through unilateral common carotid artery ligation followed by 8% oxygen for 2 h in P7 rat pups. DHF was administered intraperitoneally 2 h before and 18 h after the HI injury. A polymerase chain reaction (PCR) array was used to identify the target genes upregulated after the DHF treatment, which was then confirmed with quantitative real-time reverse transcriptase PCR and a western blot. Effects of the downstream mediator of DHF were assessed using an intravitreal injection of neutralizing antibody 4 h after DHF administration (24 h after HI). Meanwhile, the target protein was injected into the vitreous 24 h after HI to validate its protective effect when exogenously supplemented. We found that systemic DHF treatment after HI significantly increased the expression of the artemin (*ARTN*) gene and protein at P8 and P10, respectively. The neuroprotective effects of DHF were inhibited after the ARTN protein blockade, with an increase in neuroinflammation and astrogliosis. ARTN treatment showed long-term protection against HI injury at both the histopathological and functional levels. The neuroprotective effects of ARTN were related to a decrease in microglial activation at P17 and attenuation of astrogliosis at P29. ARTN enhances phosphorylation of RET, ERK, and JNK, but not AKT or p38 in the immature retina. Altogether, these results suggest that the neuroprotective effect of a TrkB agonist is partially exerted through a mechanism that involves ARTN because the protective effect is ameliorated by ARTN sequestration. ARTN treatment after HI injury protects the immature retina by attenuating late neuroinflammation and astrogliosis in the immature retina relating to the ARTN/RET/JNK/ERK signaling pathway. ARTN may be a strategy by which to provide long-term protection in the immature retina against HI injury.

## Introduction

With advances in perinatal care, the survival rates of infants with hypoxic-ischemic encephalopathy have increased (Lee et al., 2013). Up to 60% of infants who survive have severe disabilities, including intellectual disability, epilepsy, and cerebral palsy (Pierrat et al., 2005); however, hypoxic-ischemia (HI) is also a major cause of acquired visual impairment in children from developed countries (Tinelli et al., 2020). Although cortical visual dysfunction is an important cause of visual impairment, our previous studies demonstrated that the immature retina is also susceptible to HI injury (Huang et al., 2012, 2015). Compared with adult rodents, HI has been shown to cause more rapid and extensive damage of the retina at both the histopathological and functional levels in rat pups, involving prominent neuroinflammation with astrogliosis and caspase-dependent apoptotic neuronal death (Huang et al., 2012, 2015).

The neuroprotective role of brain-derived neurotrophic factor (BDNF) in the retina has been extensively tested over the past two decades (Afarid et al., 2016). Through the activation of tropomyosin receptor kinase B (TrkB), BDNF leads to increased neurogenesis, neuronal survival, and differentiation (Harada et al., 2011). However, the use of BDNF as a treatment for retinal degeneration has not been successful due to the challenges related to sustaining adequate therapeutic levels (Daly et al., 2018). The use of BDNF mimetics has thus been investigated as an alternative treatment against neuronal injury (Jang et al., 2010). In a previous study, we showed that in rat pups, the systemic administration of 7,8-dihydroxy-avone (DHF), a selective TrkB agonist, provided long-term protection against retinal HI injury at both the histological and functional levels and was related to both decreased astrogliosis and increased neurogenesis (Huang et al., 2018). To elucidate the subsequent gene expression involved in the TrkB-mediated neuroprotection, we used a quantitative real-time polymerase chain reaction (qRT-PCR) array and found that *artemin* was selectively upregulated after DHF treatment in the immature retina with an HI injury (see section “Results”).

Artemin (ARTN) is a member of the glial cell line-derived neurotrophic factor (GDNF) family of ligands (GFLs, including GDNF, neurturin, ARTN, and persephin), which form ternary complexes with the GDNF family receptor (GFRα). Assembling of the GFL-GFRα-RET (tyrosine kinase receptor) complex triggers the dimerization of RET, leading to autophosphorylation of specific tyrosine residues in its intracellular domain and subsequent activation of different intracellular signal cascades. These include AKT, ERK, JNK, P38, and Src, which are involved in the regulation of cell survival, neurite outgrowth, and synaptic plasticity (Wong et al., 2015; Nencini et al., 2018). Previous studies have demonstrated the neuroprotective effects of GDNF against HI brain damage in neonatal rats (Ikeda et al., 2000). However, our array data showed significantly elevated *ARTN* mRNA instead of *GDNF* in DHF-treated HI retinas (see section “Results”). In the eye, ARTN is primarily expressed in the retina, and provides neuroprotection in cases of retinal degeneration or after axotomy (Hauck et al., 2006; Omodaka et al., 2014). Accumulating evidence indicates that ARTN plays a critical role in the adaptability of cancer cell populations to hostile challenges such as chemotherapeutics and ionizing radiation (Ding et al., 2014). The adaptive response involves hypoxia-induced ARTN, which promotes the epithelial-mesenchymal transition and decreased apoptosis (Hezam et al., 2018). However, it remains to be determined whether ARTN can rescue immature retina after HI injury, as well as the mechanisms involved.

## Materials and Methods

### Animals

This study was approved by the Animal Care Committee and the Ethics committee of Chang Gung Memorial Hospital in Kaohsiung. Ten to twelve Sprague-Dawley rat pups per dam were used and housed with a 12/12 h light/dark schedule in a temperature- and humidity-controlled colony room. The pups were housed with their dams until weaning at postnatal (P) day 21 and then housed in groups of 4–5 per cage.

### Hypoxic-Ischemia Eye Injury

At P7, the animals were anesthetized with 2.5% halothane (balance, room air), and the right common carotid artery was surgically exposed and permanently ligated. After surgery, the pups were returned to the dam for 1 h, then placed in air-tight containers through which humidified 3 L/min 8% oxygen (balance, nitrogen) was maintained for 2 h (Huang et al., 2012). The sham controls underwent anesthesia and surgical exposure but did not receive artery ligation and were not placed in a hypoxic chamber.

### Systemic DHF Treatment

Two hours before and 18 h after the induction of HI, the rat pups were injected intraperitoneally with either DHF (5 mg/kg; Tokyo Chemical Industry Co., Tokyo, Japan) or dimethyl sulfoxide (DMSO; 10%; Sigma-Aldrich Corp., St. Louis, MO, United States).

### PCR Array

The Rat Neurogenesis RT^2^ Profiler ^TM^ PCR Array (Qiagen, Catalog # PARN-404Z, MD, United States), which consisted of primers for 84 genes related to neurogenesis and neural stem cells was used for the gene expression analysis. The rats treated with either DHF or DMSO were sacrificed, and total RNA was prepared from the retinas at P10 and immediately frozen at −70°C. Aliquots of 1 μg RNA per retina were reverse-transcribed using a RT^2^ First Strand Kit (Qiagen). The complementary DNA (cDNA) was mixed with SYBR Green (Qiagen) into the array plates, and cycling was performed according to the manufacturer’s protocol. The data obtained from the array were normalized using multiple housekeeping genes and analyzed by comparing 2^–Δ*Ct*^ of the normalized data. Fold changes were calculated relative to the retinal extracts from HI animals treated with DHF and DMSO. The results were confirmed using a quantitative real-time reverse transcriptase polymerase chain reaction analysis on the individual samples for genes that showed the strongest upregulation and downregulation.

### Quantitative Real-Time Reverse Transcriptase Polymerase Chain Reaction

Retinas were dissected and ground with a mortar and pestle in liquid nitrogen under RNase-free conditions. Total RNA was extracted using TRIzol reagent (Invitrogen, Carlsbad, CA, United States). Aliquots of 5 μg total RNA were reverse-transcribed to cDNA using SuperScript III Reverse Transcriptase (Invitrogen). The cDNA was amplified by PCR using the following gene-specific primers: *ARTN*, 5′-CAGAGCCTGGAAAGATGACC-3′ (forward) and 5′-AGAGCTGGGATCCATGAACA-3′ (reverse); and glyceraldehyde 3-phos-phate dehydrogenase (*GAPDH*), 5′-TCTTGTGCAGTGCCAGCCTC-3′ (forward) and 5′-GTCACAAGAGAAGGCAGCCCTGG-3′ (reverse). The template was amplified at 95°C for 5 min, followed by 45 cycles of PCR at 95°C for 10 s, 60°C for 20 s, and 72°C for 20 s using the LightCycler^®^ 480 SYBR Green I Master (Roche, Indianapolis, IN, United States) and LightCycler^®^ 480 instrument (Roche) for analyzing the *ARTN* and *GAPDH*. The C_ t_ assigned as the beginning of the logarithmic amplification was computed using the equipment’s software program (Roche). The relative expression level was defined as 2^–Δ*Ct*^, where ΔC_t_ = C_t target gene_ – C_t β –actin_. The fold changes in mRNA expression were defined as 2^–ΔΔ*Ct*^, where ΔΔC_t_ = ΔC_t treatment_ – ΔC_t vehicle_.

### Investigation of the Effects of Artemin

An evaluation of whether the protective effect of DHF comes from upregulating endogenous ARTN, intravitreal injection of either the ARTN-neutralizing antibody (ARTN Ab, 1 μg; R&D systems, Minneapolis, MN, United States) (DeBerry et al., 2015), or phosphate buffered saline (PBS) was performed at post-HI 24 h, which was 6 h after the DHF treatment. To assess the effect of exogenous ARTN in HI injured retinas, either ARTN (1 μg, Peprotech, Rocky Hill, NJ, United States) (Omodaka et al., 2014) or H_2_O was administered by intravitreal injection at post-HI 24 h. The animals received intraperitoneal injection of 5-bromo-2′-deoxyuridine (BrdU; 100 mg/kg; Sigma-Aldrich Corp.) for three consecutive days from P8 to P10 for the purpose of identifying cell proliferation.

### Functional Evaluation of the Retina Using Electroretinography

At P22 and P29, full-field scotopic flash electroretinograms (ERGs) (RETIport ERG; Roland Consult, Brandenburg, Germany), were recorded from both eyes of the rat pups, as previously described (Huang et al., 2012). Briefly, the pupils were topically dilated with 1% Tropicamide (Mydriacyl, Alcon, Puurs, Belgium) and 1% cyclopentolate (Cyclogyl, Alcon, Puurs, Belgium), then the eyes were dark-adapted for 1 h before performing ERG. The animals were sedated using intramuscular injections of a mixture of Rompun (10 mg/kg; Bayer Korea, Seoul, South Korea) and Zoletil-50 (25 mg/kg; Virbac, Carros, France); then, a standard white flash on a dark background scotopic 0-dB ERG was recorded. The stimulus luminance was 3 cds/m^2^ with a duration of 10 ms. Responses from 20 identical flashes applied at 10-s intervals were averaged (Huang et al., 2015).

### Histological Assessment of Retinal Injury

Paraffin sections of the retina were dewaxed, hydrated through graded concentrations of alcohol, and placed in phosphate-buffered saline. Cryosections were prepared after fixation in 4% paraformaldehyde and dehydration in a sucrose gradient. Eye blocks were cut at 10 μm. Two sections per retina were randomly selected for hematoxylin and eosin staining. Images were acquired using a light microscope (Nikon, Tokyo, Japan). Retinal damage was quantified within central retinal areas (100–200 μm from the optic disk), as described in our prior study (Huang et al., 2012) by assigning different grades: grade 0, preserved retinal ganglion cell (RGC) and all retinal layers comparable to the sham control; grade 1, moderate decrease in RGC counts and thickness of the inner plexiform layer (IPL); grade 2, complete loss of RGCs and IPL (Supplementary Figure 1).

### Immunohistochemical Staining

After antigen unmasking and blocking of nonspecific sites, the sections were incubated overnight at 4°C with primary antibodies against ARTN (1:10; R&D systems), phosphorylated (p)RET (1:10; Abcam, Cambridge, United Kingdom), ED1 (1:100; Biosource, Camarillo, CA, United States), antiglial fibrillary acidic protein (GFAP; 1:200; Millipore, Temecula, CA, United States), and BrdU (1:100; Novocastra, Newcastle upon Tyne, United Kingdom) and then subsequently incubated with secondary antibodies for 60 min at room temperature. The immunoreactivity of ARTN was evaluated at a 200× magnification by calculating the integrated optical density (IOD) with ImagePro Plus 6.0 software (Huang et al., 2018). The number of ED1^+^ and Brdu^+^ cells were counted in an area of 400 × 100 μm at 200× magnification. The ameboid ED1^+^ cells were defined as reactive microglial cells. GFAP immunoreactivity was quantified by assigning different grades: grade 1, immunoreactivity in the nerve fiber layer (NFL) and around vessels; grade 2, immunoreactivity in the NFL in an outward tentacle-like pattern, extending toward the inner nuclear layer (INL); grade 3, showing occasional and grade 4, showing extensive GFAP immunoreactivity extending from the NFL to the outer nuclear layer (ONL; Huang et al., 2018).

### Western Blot Analysis

Retinas were homogenized, and 40 μg samples were resolved using a 10% sodium dodecyl sulfate polyacrylamide gel electrophoresis, after which they were blotted to nitrocellulose membranes. Membranes were blocked with 5% non-fat dry milk, incubated with primary antibodies and horseradish-conjugated secondary antibodies, and the signal was visualized with enhanced chemiluminescence. The following primary antibodies were used: anti-ERK (1:10,000; Cell Signaling Technology, Danvers, MA, United States), anti-pERK (1:2000; Millipore), anti- c-Jun N-terminal kinase (JNK, 1:2,000; Cell Signaling Technology), anti-pJNK (1:1,000; Cell Signaling Technology), anti-p38 (1:5,000; Abcam), anti-pp38 (1:10,000; Abcam), anti-Akt (1:10,000; Cell Signaling Technology), and anti-pAkt (1:1,000; Cell Signaling Technology). After the densitometric analysis, data were normalized against GAPDH (Millipore), and the ratio of protein expression in the treated eyes to the sham controls was calculated.

### Statistics

Statistical analyses were performed using a 1-way ANOVA or a Kruskal–Wallis test using GraphPad Prism 4 software (GraphPad, San Diego, CA, United States). Data were presented as mean ± standard error, and *p* values of <0.05 were considered statistically significant.

## Results

### Systemic DHF Treatment Increases the Expression of ARTN After HI Injury in Immature Retinas

**FIGURE 1:**
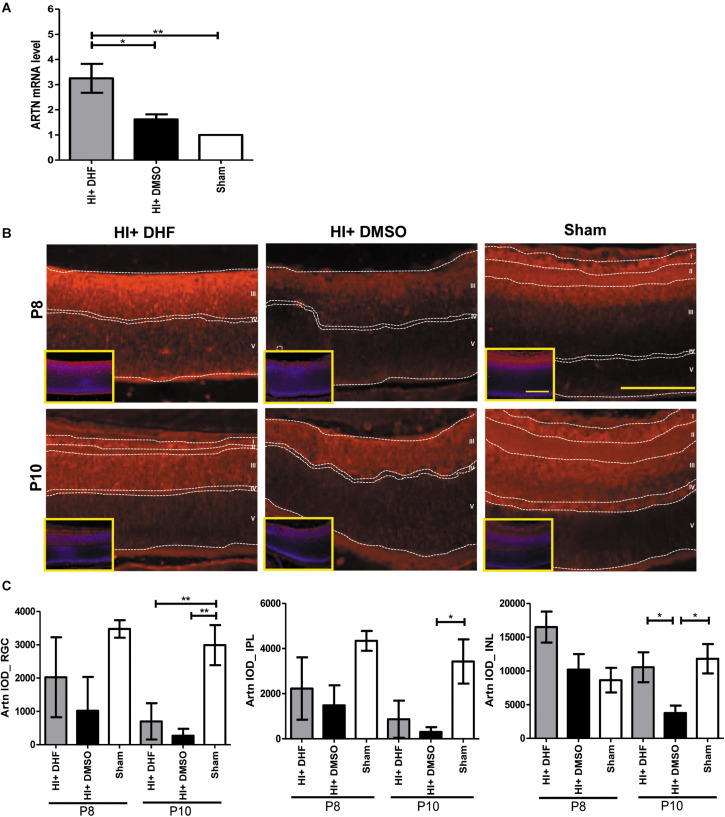
Systemic TrkB agonist- DHF increases ARTN expression in the HI Injured immature retina. **(A)** At P8, the ARTN mRNA level significantly increased in the DHF-treated HI group as compared with the DMSO-treated HI and sham control groups. **(B)** The immunohistochemical stain showed that ARTN protein was expressed in the retina ganglia cells (RGC, layer I), inner plexiform layer (IPL, layer II) and the upper part of inner nuclear layer (INL, layer III) of the sham controls at P8. The ARTN protein expression further extended to outer plexiform layer (OPL, layer IV) in sham controls at P10. There was almost complete loss of layers I and II but preserved layers IV and V (outer nuclear layer, ONL) in both HI groups at P8. The DMSO-treated HI group had decreased ARTN immunostaining in layer III at P8 and P10. In contrast, the DHF-treated HI group had relatively preserved ARTN protein expression in the whole layer III at P8 and P10. The insets showed the nuclear counterstaining with DAPI (blue). **(C)** The group data showed that there was significant difference in the ARTN immunoreactivity of the RGC layer between the sham controls and both the HI group at P10. The DHF-treated HI group had significantly increased ARTN protein expression in the INL than the DMSO-treated HI group at P10. There was no significant difference of ARTN immunoreactivity in the INL between the sham controls and the DHF-treated HI group. Scale bars = 100 μm in figures and insets; *n* = 4 to 10 per group in each time point, power = 0.8, *p < 0.05, **p < 0.01.

Previously we showed that systemic DHF treatment was able to protect the immature retina against HI injury (Huang et al., 2018). To identify the mediators for the HI protective effects of DHF treatment in the immature retina, a PCR array was performed at P10, and it was found that the expression of ARTN gene in the DHF-treated HI group was 2 times higher than that in the DMSO-treated HI group. In contrast, other neurotrophic factors, including glial cell line-derived neurotrophic factor (GDNF) and BDNF, were not significantly elevated in the DHF-treated HI group (Supplementary Table). Using a RT-PCR, we confirmed that the ARTN mRNA levels in the DHF-treated HI group were significantly higher than those in the DMSO-treated HI group and the sham controls 24 h after HI (Figure 1A). The immunohistochemical stain showed that ARTN protein was expressed in the RGC, IPL, and INL of the sham controls and the DHF-treated HI group at P8 but was decreased in the DMSO-treated HI group (Figure 1B and Supplementary Figure 2). The ARTN immunoreactivity was significantly lower in the INL of the DMSO-treated HI group compared to the DHF-treated HI group and the sham group at P10 (*p* < 0.05; Figure 1C).

### Intravitreal Injection of ARTN-Neutralizing Antibody Blocks the Long-Term Neuroprotection of DHF Against HI at Both the Functional and Histopathological Levels

**FIGURE 2:**
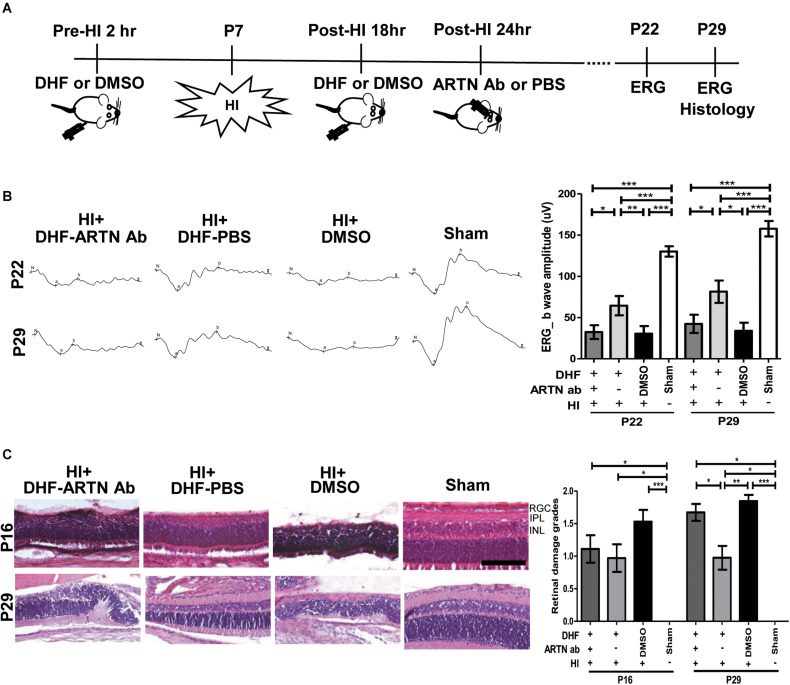
Intravitreal injection of ARTN Antibody inhibits the functional and histological protection of systemic DHF in the HI-injured retinas. **(A)** Two hours before and 18 h after the HI, either DHF or DMSO was injected peritoneally. ARTN-Antibody (Ab) or PBS was injected intravitreally at post-HI 24 h. ERG or/and histology was performed at P22 and P29. **(B)** The retinal function evaluated using ERG demonstrated markedly depressed b-wave amplitudes in the HI injured groups at P22 and P29 as compared with the sham controls. The group data showed that the b-wave amplitude in the DHF-PBS group was significantly higher than that in the DHF-ARTN Ab and DMSO-treated groups after HI at P22 and P29. **(C)** Representative retinal histologic sections showed that the number of RGCs and the thickness of IPL and INL decreased after HI at P16 and P29. The group data showed that the grades of retinal damage were significantly higher in the HI groups than in the sham controls at P16 and P29. There were no significant differences between the DHF-PBS, DMSO, and DHF-ARTN Ab groups after HI injury at P16. As compared with the DHF-PBS group, the DMSO and DHF-ARTN Ab groups exhibited increased retinal damage grades at P29. Scale bars: 100 μm; *n* = 4–13 per group in each time point, power = 0.8, *p < 0.05, **p < 0.01, ***p < 0.001.

After HI injury, endogenous ARTN was sequestered using intravitreal injections of artemin-neutralizing antibody (ARTN Ab) to determine whether the neuroprotective effects of DHF in the immature retina were mediated by artemin (Figure 2A). ERG performed at P22 and P29 showed that the a-wave (associated with rod photoreceptor activity) and b-wave (Müller glial and bipolar cells activity) amplitudes in the DHF-PBS group were relatively preserved, while in the DMSO-treated group and the DHF-ARTN Ab group, the amplitudes of the a-wave and b-wave were markedly decreased (Figure 2B). Group data showed that the b-wave amplitude in the DHF-ARTN Ab group was significantly lower (*p* < 0.05) than that in the DHF-PBS group but was similar to that in the DMSO-treated group (Figure 2B). There were no significant among-group differences in the a-wave amplitudes of the DHF-PBS, DHF-ARTN Ab, and DMSO-treated HI groups (data not shown).

Compatible with the functional alterations, there was almost a complete loss of the inner retinal layers, including the RGC, IPL, and INL, in addition to a partial loss of the outer retina layers, including the outer plexiform layer (OPL) and the ONL in the DMSO-treated HI group at P16 and P29 (Figure 2C). Although in general thinner than the controls, the retinal layers in the DHF-PBS group were relatively preserved after HI. In contrast, the DHF-ARTN Ab group showed severe inner retinal damages after HI, especially at P29. Semiquantitative data showed that there were no significant between-group differences in the severity of retinal damage in the DMSO-, DHF-PBS- and DHF-ARTN Ab-treated HI groups at P16. However, at P29, the DMSO- and the DHF-ARTN Ab-treated HI groups showed significantly more severe retinal damage than the DHF-PBS group (*p* < 0.05; Figure 2C). There were no significant between-group differences in the DMSO- and DHF-ARTN Ab-treated HI groups. These data suggest that the neuroprotective effects of DHF are largely mediated by ARTN and that ARTN was involved in the long-term neuroprotective effects of DHF against HI retinal injury.

### Blockade of ARTN Abolishes the Neuroprotection of DHF Through an Increase in Neuroinflammation and Astrogliosis

**FIGURE 3:**
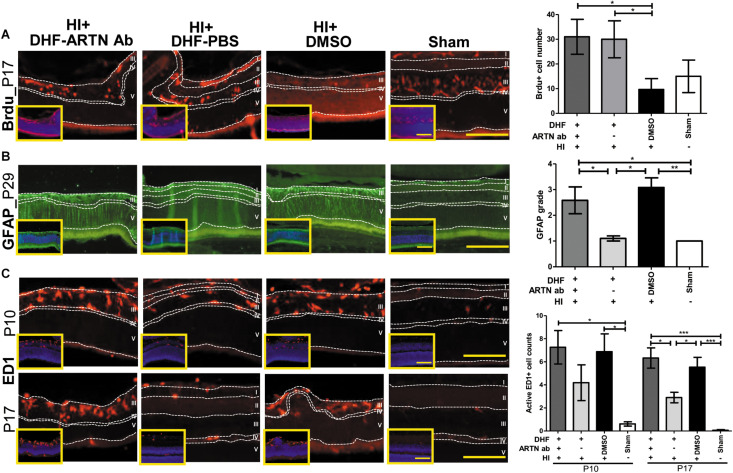
ARTN Antibody blocks the protective effect of systemic DHF treatment in HI-injured retinas by increasing neuroinflammation. **(A)** At P17, he Brdu^+^ cells localized in the retina ganglia cells (RGC, layer I), inner plexiform layer (IPL, layer II), and inner nuclear layer (INL, layer III) in sham controls. There was almost complete loss of layers I and II but preserved layers IV (outer plexiform layer, OPL) and V (outer nuclear layer, ONL) in DMSO- and DHF-ARTN Ab-treated HI groups in the representative figures. The DMSO-treated HI group had few Brdu^+^ cells in the layer III. The total Brdu^+^ cells was significantly lower in the DMSO-treated HI group as compared to in the DHF-PBS and DHF- ARTN Ab groups after HI. There was no significant difference in the DHF-PBS- and DHF- ARTN Ab-treated HI groups. **(B)** At P29, GFAP immunostaining was extensive throughout all retinal layers in the DMSO-treated and DHF-ARTN Ab groups after HI. The grades of GFAP immunoreactivity were significantly higher in the DMSO-treated and DHF-ARTN Ab groups than in the DHF-PBS and sham control groups. **(C)** The ameboid microglial cells (active ED1^+^ cells) localized in the layers I, II and III after HI at P10. The DMSO- and DHF- ARTN Ab-treated HI groups had prominent ameboid microglial cells mainly in layer III as the layers I and II were lost. The microglial cells were less active in the DHF-PBS-treated HI groups at P17. But the DMSO- and DHF-ARTN Ab-treated HI groups still had prominent ameboid microglial cells in layer III of retina. The group data showed that the active ED1^+^ cell counts were markedly increased in the DMSO- and DHF-ARTN Ab-treated HI groups as compared with the sham controls at P10 and P17. The DHF-PBS group exhibited significantly decreased active ED1^+^ cells than the DMSO and DHF-ARTN Ab groups at P17. There was no significant difference between the DMSO- and DHF-ARTN Ab-treated HI groups at P10 and P17. The insets showed the nuclear counterstaining with DAPI (blue). Scale bars: 100 μm in main figures and insets; n = 4–11 per group in each time point, power = 0.8, *p < 0.05, **p < 0.01, ***p < 0.001.

In our previous study, we also showed that the long-term neuroprotective effects of DHF were related to increased neurogenesis and decreased astrogliosis (Huang et al., 2018). In the current study, immunohistochemical staining showed that at P17, both the DHF-PBS and DHF-ARTN Ab groups had significantly increased Brdu^+^ cells in the inner retina as compared to in the DMSO-treated HI groups (*p* < 0.05; Figure 3A and Supplementary Figure 3A), suggesting that DHF promotes cell proliferation in the inner retina, but its treatment effects are not affected by the blockade ARTN protein.

The GFAP immunoreactivity, indicating the presence of astrogliosis, in the DHF-PBS group was significantly lower than that in the DMSO-treated HI group at P29. However, the DHF-ARTN Ab group had extensive GFAP immunostaining throughout all of the retinal layers, similar to the DMSO-treated HI group, which was higher than the DHF-PBS group (*p* < 0.05; Figure 3B and Supplementary Figure 3B).

At P17, reactive microglial cells, or active ED1^+^ cells, were present in the inner retina of all of the groups other than the control. However, the number of ED1^+^ cells was similar between the DMSO-treated and the DHF-ARTN Ab HI groups and was significantly higher than the number in the DHF-PBS group (*p* < 0.05; Figure 3C and Supplementary Figure 3C). These data suggest that the neuroprotective mechanisms of DHF mediated by ARTN involves modulation of astrogliosis and neuroinflammation, but not cell proliferation.

### Intravitreal Injection of ARTN Provides Long-Term Protection Against HI Injury at the Histopathological and Functional Levels in Immature Retinas

**FIGURE 4:**
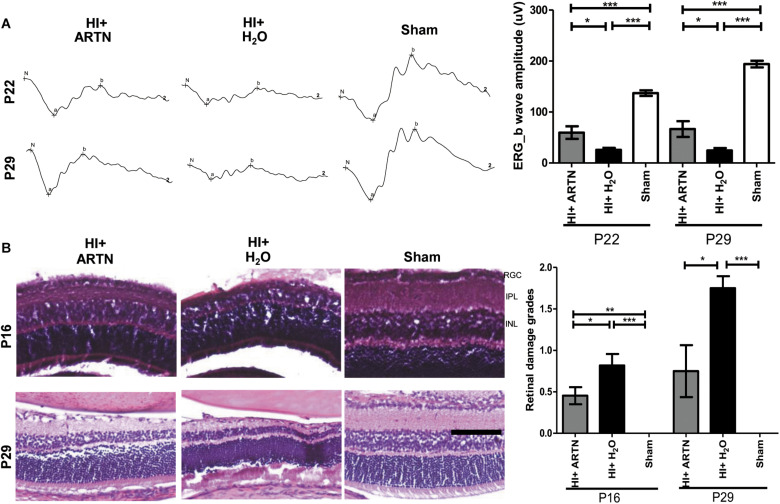
Intravitreal injection of ARTN protects immature retinas against HI injury in both functional and histological levels. **(A)** At P22 and P29, the b-wave amplitudes of ERG were significantly decreased in the HI injured groups, as compared with the sham controls. The b-wave amplitude was significantly higher in the ARTN-treated as compared to in the H_2_O-treated group after HI. **(B)** Representative retinal histologic sections showed that the number of RGCs and the thickness of IPL and INL decreased after HI. The group data showed that the retinal damage grades were markedly higher in the H_2_O-treated HI group than the ARTN-treated HI group at P16 and P29. Scale bars: 100 μm; *n* = 6–16 per group in each time point, power = 0.8, *p < 0.05, **p < 0.01, ***p < 0.001.

To verify whether ARTN provides protection against HI injury, the rat pups received intravitreal injections of either ARTN or H_2_O 24 h after HI injury. The ERG at P22 and P29 showed that the a-wave and b-wave in the ARTN-treated HI group were relatively preserved, with b-wave amplitudes that were significantly higher than those in the H_2_O-treated HI group (both *p* < 0.05; Figure 4A). In line with the functional alterations, despite the presence of damaged inner retinal layers in the ARTN-treated HI group, the damage still occurred to a lesser degree than that in the H_2_O-treated HI group and was statistically significant at both P16 and P29 (both *p* < 0.05; Figure 4B).

### ARTN Treatment Protects the Immature Retina Against HI Injury by Inhibiting Late Neuroinflammation and Astrogliosis

**FIGURE 5:**
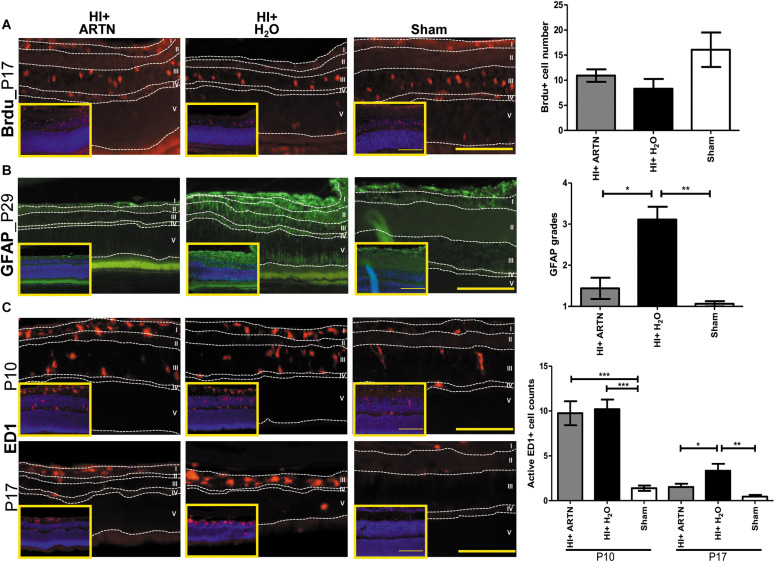
Intravitreal injection of ARTN does not increase cell proliferation but decreases neuroinflammation and astrogliosis. **(A)** The representative figures showed that the Brdu^+^ cells are mainly localized in the retinal ganglia cells (RGC, layer I) and inner nuclear layer (INL, layer III) at P17. There were no significant between-group differences in the Brdu^+^ cell counts in the ARTN- and H_2_O-treated HI groups. **(B)** At P29, the H_2_O-treated HI group had marked GFAP immunostaining extending the whole retina. The GFAP immunostaining were significantly lower in the ARTN-treated HI group as compared to in the H_2_O-treated HI group by the semiquantitative analysis. There was no significant difference between the ARTN-treated HI group and sham controls. **(C)** Active ED1^+^ cells mainly localized in the layers I, II (inner plexiform layer, IPL) and III, but not in the outer plexiform layer (OPL, layer IV) and outer nuclear layer (layer V), after HI at P10. There were persistent prominently ameboid microglial cells in the layer III of the H_2_O-treated HI groups at P17. The cell counts were significantly increased in the ARTIN-treated and H_2_O-treated HI groups than sham controls at P10. The ARTN-treated HI group had markedly decreased active ED1^+^ cells, as compared with the H_2_O-treated HI group at P17. The insets showed the nuclear counterstaining with DAPI (blue). Scale bars: 100 μm in main figures and insets; *n* = 5–16 per group in each time point, power = 0.8, *p < 0.05, **p < 0.01, ***p < 0.001.

Immunohistochemical staining showed that at P17, the number of proliferating or Brdu^+^ cells were not significantly different between the ARTN- and H_2_O-treated HI groups (Figure 5A and Supplementary Figure 4A). However, GFAP immunostaining at P29 demonstrated that ARTN treatment significantly decreased astrogliosis after HI (*p* < 0.05; Figure 5B and Supplementary Figure 4B). Both the ARTN- and H_2_O-treated HI groups showed significantly greater neuroinflammation, with increased active ED1^+^ cells, as compared to the sham controls at P10 (*p* < 0.001; Figure 5C and Supplementary Figure 4C). However, at P17, the number of active ED1^+^ cells in the ARTN-treated HI group was significantly decreased compared to the H_2_O-treated HI group (*p* < 0.05; Figure 5C and Supplementary Figure 4C), which was similar to the sham controls. These data suggest that ARTN protects the immature retina by decreasing astrogliosis and late neuroinflammation after HI.

### ARTN Treatment After HI Injury Enhances RET, ERK, and JNK Phosphorylation in Immature Retinas

**FIGURE 6:**
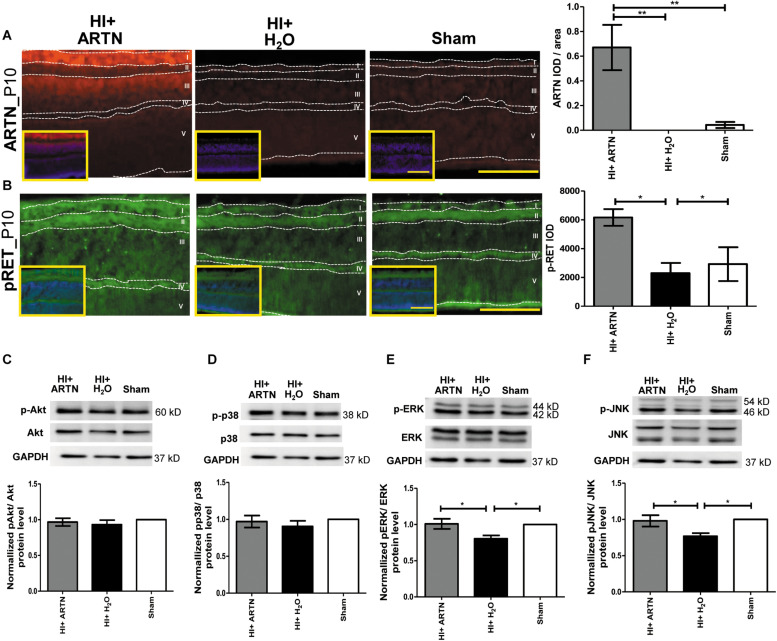
Post-treatment with ARTN enhances RET, ERK, and JNK phosphorylation in the immature retina after HI injury. **(A)** At P10, the ARTN immunostaining was prominent in the retinal ganglia cells (RGC, layer I) and inner nuclear layer (INL, layer III) of the ARTN-treated HI group. The group data showed that the immunoreactivity of ARTN was significantly higher in the ARTN-treated HI group than in the H_2_O-treated HI group and the sham controls. **(B)** Alt P10, the immunostaining of phosphorylated RET (pRET) localized in the layers I, II (inner plexiform layer, IPL), and IV (outer plexiform layer, OPL). The group data showed that the H_2_O-treated HI group had significantly decreased pRET immunostaining as compared with the ARTN-treated HI groups and the sham controls. At P10, the western blot analysis showed there were no significant differences in the **(C)** phosphorylated (p) Akt or **(D)** pp38 among the ARTN-treated HI group, H_2_O-treated HI group, and sham controls. The ARTN-treated HI group had a higher expression of **(E)** pERK and **(F)** pJNK than the H_2_O-treated HI group. Nuclear counterstaining with DAPI (blue) was shown in the inset; layer V: outer nuclear layer (ONL); Scale bars: 100 μm in main figures and insets; *n* = 4–7 per group in each time point, power = 0.8, *p < 0.05, **p < 0.01.

The ARTN-treated HI group had prominent ARTN immunostaining in the RGC and INL at P10, which indicates that ARTN continued to be expressed in the inner retina two days after the treatment. There were significant differences between the ARTN-treated HI group and the H_2_O-treated HI group and the sham controls (*p* < 0.01; Figure 6A and Supplementary Figure 5A). Immunostaining showed that the ARTN treatment after HI induces significantly increased RET phosphorylation (pRET) immunoreactivity in the RGC, IPL, and OPL (*p* < 0.05; Figure 6B and Supplementary Figure 5B).

To investigate the downstream signaling pathways of ARTN/RET, western blots were performed at P10. There were no significant differences in the total and phosphorylated p38 or AKT between the ARTN- and H_2_O-treated HI groups at P10 (Figures 6C,D). However, the ARTN-treated group exhibited significantly increased phosphorylation of ERK and JNK as compared to the H_2_O -treated HI group (both *p* < 0.05; Figures 6E,F). There were no significant differences in the total ERK and JNK expression between the ARTN- and H_2_O-treated HI groups.

## Discussion

The present study is the first to demonstrate that systemic treatment with DHF upregulates the expression of ARTN after HI injury in the immature retina. The higher expression of ARTN in the inner retina, which is more vulnerable to HI injury in an immature retina, may be one of the factors responsible for its neuroprotection. Surprisingly, the TrkB agonist triggers indirect neuroprotection through a mechanism that involves ARTN because the protection is ameliorated with ARTN sequestration. This result is consistent with our findings indicating that ARTN treatment after HI injury protects the immature retina by attenuating late neuroinflammation and astrogliosis. The ARTN/RET/JNK/ERK signaling pathway appears to be involved in the ARTN-mediated neuroprotection. We suggest that ARTN may represent a promising therapy for restoring retinal function after HI injury in neonates.

ARTN is an important mediator of various physiological and pathophysiological functions, including the development and maintenance of various neuronal populations, neurite outgrowth, and nerve regeneration (Wong et al., 2015). ARTN has been used in a clinical trial as a treatment for depression based on its neuroplasticity effects (Di Cesare Mannelli et al., 2011). There is increasing evidence that ARTN plays an important role in the adaptation of cancer cell populations to challenges that are not conducive to survival. The adaptive response involves hypoxia-induced ARTN promotion of epithelial-mesenchymal transition and decreased apoptosis (Ding et al., 2014; Hezam et al., 2018). Recent evidence suggests that ARTN plays a bi-directional role in the modulation of neuropathic pain and inflammation (Ikeda-Miyagawa et al., 2015). ARTN is increased by inflammation and mediates nociceptive signaling in both humans and animals (Thornton et al., 2013; Ikeda-Miyagawa et al., 2015; Nencini et al., 2018). Anti-ARTN Ab treatment has been shown to inhibit ARTN/RET/ERK activation and block capsaicin-induced calcitonin gene-related peptide secretion *in vitro* (Jeong et al., 2008). Conversely, a phase 2 clinical trial showed evidence of pain relief by ARTN in patients with lumbosacral radiculopathy (Backonja et al., 2017). Although the exact mechanisms underlying the regulation of inflammation by ARTN require further investigation, the findings of this study provide novel evidence suggesting that ARTN may protect the immature retina via its anti-inflammation action, likely through RET/JNK/ERK signaling pathway.

Post-ischemic neuroinflammation in the immature central nervous system is a key pathophysiological factor in the development of HI-related injury (Borjini et al., 2019). It is highly likely that this secondary inflammation augments damage in the early phase of evolving cell death (Hagberg et al., 2015). The downstream mediators of inflammation-induced injuries include induction of immune mediators, reactive oxygen and nitrogen species, excitotoxicity, mitochondrial impairment, and reduced vascular integrity (Hagberg et al., 2015). Microglial infiltration and astrogliosis has shown to potentially persist up to 21 days or 2 months after HI insult in animal and human studies, respectively (Galinsky et al., 2017; Galinsky et al., 2018). We also found that microglial activation in the inner retina from 6 h post-HI reached a peak level on P9, decreased from P14, and persisted to P33 in neonates. In contrast, the retinal Müller glial cells was shown to undergo reactive astrogliosis throughout all the ipsilateral retina layers from P21 to P60 after HI (Huang et al., 2012). Our data showed that ARTN treatment significantly attenuated microglial activation at P17 but not at P10. It seems that ARTN plays an important role in modulating late inflammation after HI injury. ARTN was also found to significantly decrease late astrogliosis at P29. While the mechanisms need to be further investigated, these data imply that supplementing or enhancing levels of ARTN make it possible to block the secondary or chronic inflammation caused by microglia and retinal Müller glial cells activation, which may be a viable therapeutic target leading to improvements in neurodevelopmental outcomes after HI injury.

The BDNF/TrkB signaling pathways and their requirements in the neuroprotection and neuron development processes are well-established, but little is known about how ARTN, one of the GFLs, induces retinal protection. BDNF protects injured RGCs both *in vitro* and *in vivo*, acting directly on the RGCs that express TrkB (Afarid et al., 2016). Conversely, it has been demonstrated that GFLs do not enhance the survival of RGCs *in vitro*, despite the fact that they enhance RGC survival *in vivo* (Koeberle and Ball, 2002). These results suggest that GFLs do not act directly on RGCs to increase cell survival *in vivo*. However, they reduces glutamate-mediated excitotoxicity in axotomized RGCs related to increasing the expression of the glutamate/aspartate transporter-1 in retinal Müller glial cells and astrocytes (Harada et al., 2011). Previous findings are, in general, consistent with the findings of the present study, in which it was found that increased ARTN levels, either induced by DHF treatment or exogenously supplemented by intravitreal administration, protect the immature retina against HI injury by ameliorating the activation of microglia and astrocytes, but this increase does not promote cell regeneration.

Recent evidence suggests that neurotrophic rescue of retinal neurons can be indirect, mediated by the interaction of other neurotrophic factors with glial cells, which in turn release secondary factors acting directly on neurons (Hauck et al., 2006). BDNF has no direct effect on isolated photoreceptor cells. Thus, it may protect photoreceptors, at least partly, through retinal Müller glial cells. BDNF-treated cultured retinal Müller glial cells was found to express BDNF, GDNF, and basic fibroblast growth factor (bFGF), in turn protecting RGCs against glutamate toxicity (Harada et al., 2011). Retinal Müller glial cells responded to Neurotrophin-3 or nerve growth factor (NGF), respectively, by increasing or decreasing their production of bFGF, which in turn resulted in either the protection or increased apoptosis of photoreceptor cells (Harada et al., 2015). We previously demonstrated that systemic DHF treatment does not prevent early neuronal apoptosis, but rather enhances the proliferation of retinal Müller glial cells and bipolar cells, thereby restoring retinal function after HI injury in rat pups (Huang et al., 2018). The present study further shows that the blockade of endogenous ARTN protein abolishes the long-term neuroprotective effects of DHF through increasing late neuroinflammation and astrogliosis. These data suggest that TrkB signaling involves not only RGCs but also retinal Müller glial cells, astrocytes, and microglia leading to neuroprotection. Therefore, a promising neuroprotective strategy may involve not only promoting neuronal survival but also may involve the cross-talks of neurotrophic factors with which the microglia, retinal Müller glial cells, and inner retinal neurons communicate during HI retinal injury.

We found that ARTN post-treatment activates both ERK and JNK signaling pathways in the immature retina during HI injury. The GFLs and GFR communications in the retina activate distinct signaling cascades, including ERK, JNK, and AKT, but with different time courses that are often involved in complex cross-talk on multiple levels (Hauck et al., 2006). These signaling pathways are involved in various cellular processes, including cell proliferation, differentiation, senescence, and apoptosis. The coordination between these pathways determines a cell’s fate. Our previous data showed that ERK activation is pivotal for the long-term neuroprotective effects of DHF treatment. ERK signaling mediates the effects of TrkB on retinal neuron proliferation and transdifferentiation (Huang et al., 2018). Conversely, JNK contributes to the apoptotic response of neuronal cells, and JNK-dependent apoptosis can be suppressed by the activation of ERK (Clark, 2018). However, recent studies indicate that periostin, an adhesion molecule, enhances the migration and differentiation of mesenchymal stem cells via the JNK signaling pathway under inflammatory conditions (Tang et al., 2017). Although the exact mechanism underlying the neuroprotection of ARTN needs further clarification, the findings of this study indicate that the intracellular signaling dynamics between ERK and JNK may play a role in the inflammatory process in HI retinal injury.

The advent of therapeutic hypothermia for neonatal HI injury made it possible to intervene and alter the course of this disease with improved rates of infant survival (Higgins et al., 2011). However, 40% of infants with severe HI injury still suffer from significant neurologic disability (Shankaran, 2015). The combination of perinatal infection and HI insult causes greater brain injury and poorer response to therapeutic hypothermia (Osredkar et al., 2014). These concerns highlight the need for adding alternative therapeutic strategies, especially those targeting neuroinflammation, beyond hypothermia therapy for HI injury. Our data suggest that ARTN is a promising candidate since it provides long-term protection to the immature retina against HI injury by ameliorating late neuroinflammation.

## Data Availability Statement

The original contributions presented in the study are included in the article/Supplementary Material, further inquiries can be directed to the corresponding author/s.

## Ethics Statement

The animal study was reviewed and approved by Animal Care Committee and the Ethics Committee of Chang Gung Memorial Hospital in Kaohsiung.

## Author Contributions

H-MH designed the research, planned and performed the experiments, analyzed the data, and drafted the manuscript. C-CH designed a part of the research and collaborated in performing the experiments. LY-CP analyzed the data and revised the manuscript. Y-CC designed the research project, analyzed the data, and revised the manuscript. All authors read and approved the final manuscript.

## Conflict of Interest

The authors declare that the research was conducted in the absence of any commercial or financial relationships that could be construed as a potential conflict of interest.

## Supplementary Material

Supplementary Figure 1 Grading of retinal damage. Grade 0, preserved RGCs and all retinal layers comparable to sham control; grade 1, moderate decreases in the RGC counts and thicknesses of the IPL; grade 2, complete loss of RGCs and IPL. Scale bars: 100 μm.

Supplementary Figure 2 Systemic TrkB agonist- DHF increases ARTN expression in the HI Injured immature retina. In the DHF-treated HI and sham control groups, double staining of ARTN (red) and DAPI (blue) showed the immunoreactivity of ARTN was prominent in the RGC, IPL, and INL at P8, and the expression decreased at P10. Scale bars: 100 μm.

Supplementary Figure 3 ARTN Antibody blocks the protective effect of systemic DHF in HI-injured retinas by increasing neuroinflammation. Nuclear counterstaining with DAPI (blue) showed **(A)** most of the Brdu^+^ cells (red) localized in the RGCs, IPL, and INL at P17; **(B)** GFAP immunostaining (green) was quite strong and extensive throughout all retinal layers in the DMSO-treated and DHF-ARTN Ab groups after HI at P29; **(C)** active ED1^+^ cells (red) localized in the RGC, IPL, and INL after HI at P10 and P17. Scale bars: 100 μm.

Supplementary Figure 4 Intravitreal injection of ARTN does not increase cell proliferation but decreases neuroinflammation and astrogliosis. Nuclear counterstaining with DAPI (blue) showed **(A)** most of the Brdu^+^ cells (red) are localized in the RGC and INL at P17; **(B)** the GFAP immunostaining (green) was quite strong and extensive throughout all retinal layers in the H_2_O-treated group as compared to in the ARTN-treated group at P29; **(C)** active ED1^+^ cells (red) localized in the IPL and INL after HI at P10 and P17. Scale bars: 100 μm.

Supplementary Figure 5 Post-treatment with ARTN enhances RET phosphorylation in the immature retina after HI injury. Nuclear counterstaining with DAPI (blue) showed **(A)** the ARTN immunostaining (red) was prominent in the RGC and INL of the ARTN-treated HI group at P10; **(B)** the immunostaining of phosphorylated RET (pRET, green) localized in the RGC, IPL, and OPL at P10. Scale bars: 100 μm.

## Abbreviations

ARTN: artemin
ARTN Ab: ARTN-neutralizing antibody
BDNF: brain-derived neurotrophic factor
DHF: 7,8-dihydroxyavone
DAPI: 4’,6 − diamidino-2-phenylindole
ERG: electroretinography
ERK: extracellular signal-regulated kinase
GDNF: glial cell line-derived neurotrophic factor
GFAP: anti-glial fibrillary acidic protein
GFLs: GDNF family of ligands
GFRα: GDNF family receptor
HI: Hypoxic-ischemia
hr: hour
INL: inner nuclear layer
IOD: integrated optical density
i.p.: intraperitoneally
IPL: inner plexiform layer
i.v.i: intravitreal injection
JNK: c-JunN-terminal kinase
NFL: nerve fiber layer
ONL: outer nuclear layer
P: postnatal
RGC: retinal ganglion cell
TrkB: tropomyosin receptor kinase B.
