# Supplementary material for: Artemin Is Upregulated by TrkB Agonist and Protects the Immature Retina Against Hypoxic-Ischemic Injury by Suppressing Neuroinflammation and Astrogliosis
Source: Front Mol Neurosci. 2021 Apr 12;14:645000. doi: 10.3389/fnmol.2021.645000 (PMC8072488; doi:10.3389/fnmol.2021.645000)

Supplementary Figure S1.

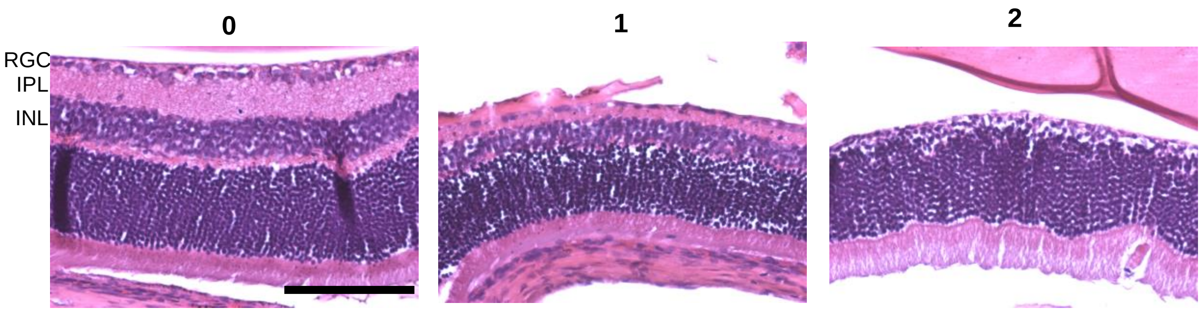

Supplementary Table. Fold changes in the expression of genes associated growth factors between DHF- and DMSO-treated retinas at P10

Gene Symbol	Description	Fold change
Gdnf	Glial cell derived neurotrophic factor	-1.257
Bdnf	Brain-derived neurotrophic factor	-1.2058
S100a6	S100 calcium binding protein A6	1.014
Ndp	Norrie disease (pseudoglioma) (human)	1.1251
Vegfa	Vascular endothelial growth factor A	1.1329
Gpi	Glucose phosphate isomerase	1.1487
Nrg1	Neuregulin 1	1.1567
Ptn	Pleiotrophin	1.2483
Fgf2	Fibroblast growth factor 2	1.3379
Mdk	Midkine	1.3379
Egf	Epidermal growth factor	1.4142
Artn	Artemin	2.1735

Supplementary Figure S2.

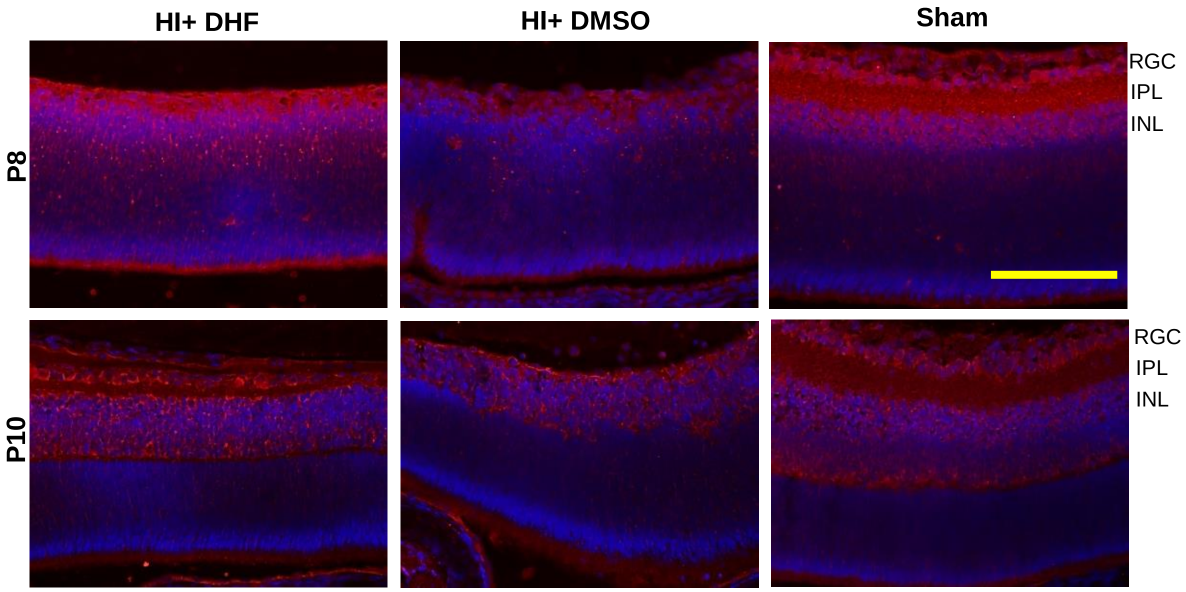

Supplementary Figure S3.

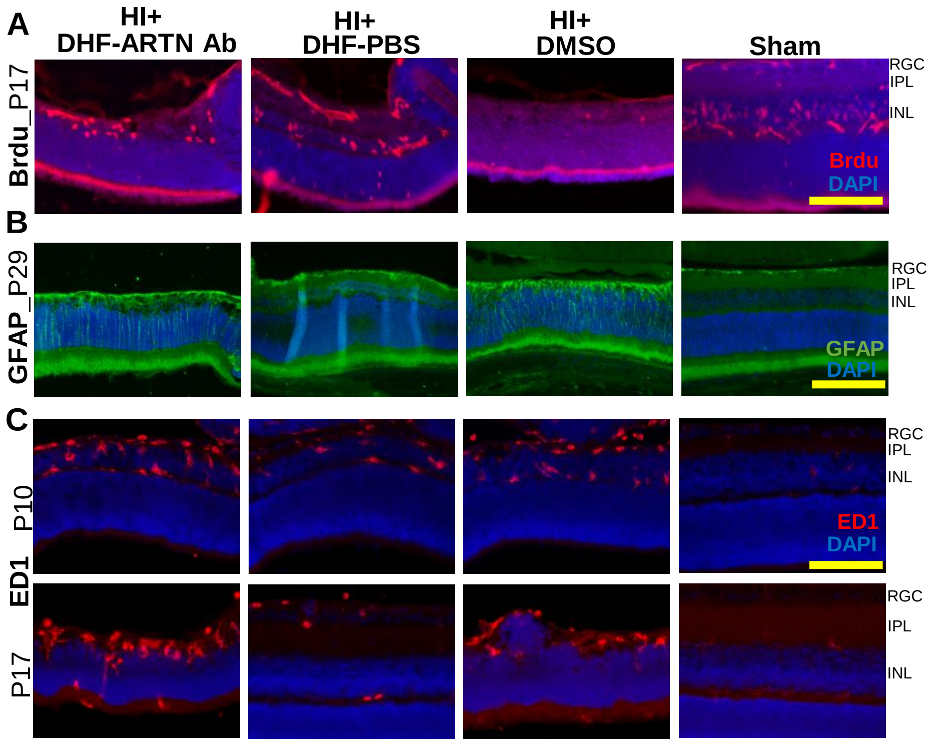

Supplementary Figure S4.

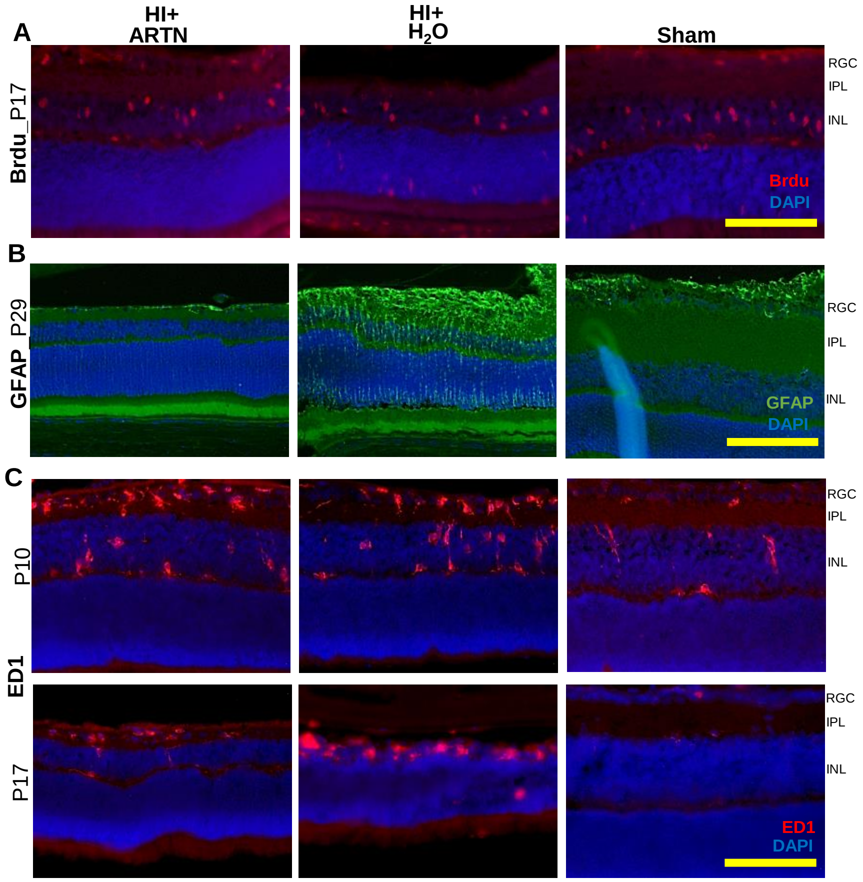

Supplementary Figure S5.

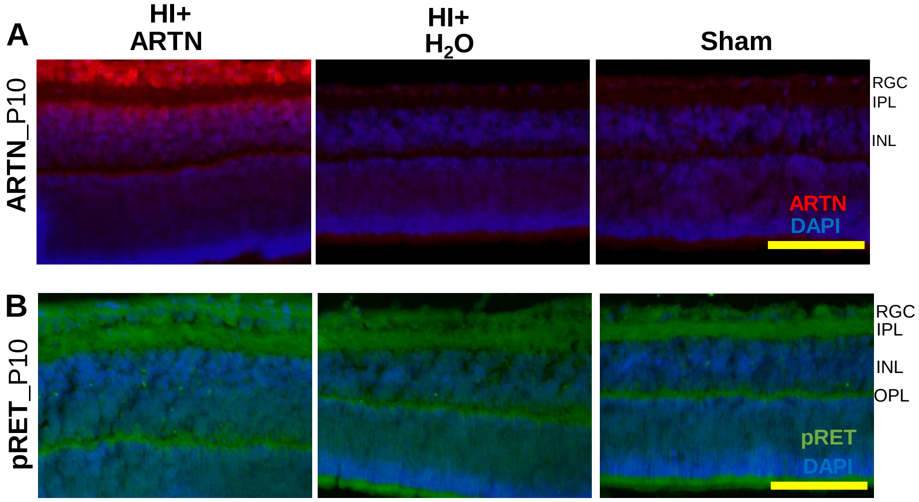

Supplement: Supplementary Figure 1 — Grading of retinal damage. Grade 0, preserved RGCs and all retinal layers comparable to sham control; grade 1, moderate decreases in the RGC counts and thicknesses of the IPL; grade 2, complete loss of RGCs and IPL. Scale bars: 100 μm. [file Data_Sheet_1.PDF]
